# The development and evaluation of a microbiota education programme intended for women

**DOI:** 10.1590/1806-9282.20242017

**Published:** 2025-08-08

**Authors:** Havva Reyhan Simsek Sira, Ozgenur Hacioglu

**Affiliations:** 1 Kırklareli University, Institute of Health Sciences, Department of Midwifery – Kırklareli, Turkey.; 2 Kırklareli University, Faculty of Health Sciences – Kırklareli, Turkey.

**Keywords:** Microbiota, Education, Women, Health

## Abstract

**OBJECTIVE:**

Findings in the literature indicate that women often have incorrect or incomplete knowledge about the concepts of microbiota, microbiome, probiotics, prebiotics, and their relationship with women's health, pregnancy, childbirth methods, and breast milk. In this regard, designing educational programs that contribute to preventing reproductive system infections, achieving positive fertility outcomes, and supporting the health processes of women and newborns through the microbiota of the female body can contribute to the current situation. In line with this aim, in this study, the development and evaluation of a microbiota education program for women were conducted.

**METHODS:**

The research was conducted with 151 female participants in a pre-test and post-test, single-group, quasi-experimental design. The trainings were conducted for 2 days, with a total of 4 h for each group, 2 h each day. The microbiota knowledge test applied before the training was repeated as a post-test after 4 weeks. SPSS 25.0 was used for data analysis.

**RESULTS:**

It was determined that there was a statistically significant difference between the pre-training and post-training knowledge scores of the participants, with the post-training knowledge scores being higher than the pre-training scores (p<0.05). It was found that there was an increase in intra-group means for age, education level, occupation, number of pregnancies, number of births, and method of delivery after the training.

**CONCLUSION:**

As a result of the evaluations, it was concluded that the microbiota education program developed and implemented for women addressed all female participants.

## INTRODUCTION

Microbiota refers to the microorganisms present in humans. The genome of these microorganisms is called the microbiome. Microbiota is a community of living organisms consisting of bacteria, viruses, protozoa, and other microorganisms that inhabit various areas of the human body, such as the oral cavity, skin, respiratory tract, intestines, and vagina^1,2^.

The human microbiota begins to develop in the womb. The microbiota that starts to form during the intrauterine period grows and changes in relation to human health as the individual ages. Previously, it was believed that the womb was sterile; however, this notion has changed with the detection of various microorganisms in the placenta, umbilical cord, amniotic fluid, and meconium^2^. It is known that the placenta, which facilitates blood flow to the baby, contains a small amount of a rich microbiota gene. Factors affecting the development of the microbiota include genetic factors, age, mode of birth, nutrition, antibiotic use, lifestyle, and environmental factors^1^.

Recent studies have emphasized the importance of the microbiota in preventing reproductive system infections and achieving positive fertilization outcomes, highlighting its significance for women's health. Concurrently, the effects of microbiota on mode of birth and neonatal health are among the most reported topics^2–4^. In this regard, recognizing the microbiota and understanding the factors that influence it is seen as a practice that can contribute to supporting women's and neonatal health^5–7^. The aim of this study was to implement an educational program on microbiota and women's health, which could be suitable for different education levels, and to assess the knowledge levels of participating women before and after the training.

## METHODS

### Study design and participant recruitment

The study was designed as a single-group quasi-experimental study with pre-tests and post-tests. A total of 151 people who agreed to attend the training regularly participated in the training program. Participants received training for a total of 4 h over 2 weeks, with 2 h of instruction each week.

### Instrument

Participant introductory characteristics questionnaire and microbiota knowledge form (the test form used in pre-tests and post-tests) were used as data collection tools. The microbiota knowledge form was administered to participants again as a post-test 4 weeks after the training.

Microbiota Knowledge Form: This form contains 20 questions related to the topics covered in the training, and participants were asked to respond with "True" or "False." Each correct answer earned the participant 5 points, while no points were awarded for incorrect answers. Participants who answered all questions correctly received a total score of 100 points. Participants answered the microbiota knowledge form as both a pre-test and a post-test, after which the pre-test and post-test scores were compared.

The training program includes seven topic areas: introduction to microbiota, human microbiota (women's health, vaginal microbiota), fertilization, mode of birth, pregnancy, breastfeeding, and probiotics.

### Ethical aspect

Before starting the research, necessary permissions were obtained from the Ethics Committee of Kirklareli University Institute of Health Sciences (Protocol No: PR0418R0, Decision No: 7, Decision Date: 17.10.2022) and from the Kirklareli Provincial Health Directorate (Decision No: 33, Decision Date: 26.12.2022). All stages of the study were conducted in accordance with the Declaration of Helsinki.

### Statistical analysis

The data obtained from the research were analyzed using the free trial version of SPSS Statistics (Statistical Package for Social Sciences) for Windows 25.0. Descriptive statistical methods were used to evaluate the data. Non-parametric tests were used for the evaluation of the variables. The analysis results were evaluated at a significance level of p<0.05.

## RESULTS

A total of 151 women participated in the research, and their distribution according to demographic characteristics is shown in Table 1.

**Table 1:** Distribution of participants according to demographic characteristics.

Variables		n	%
Age ( $\bar{\text{X}}$ ±SD, 27.22±5.63)	Under 27 years	71	47
	27 years and older	80	53
Education level	Primary School	1	0.7
	High school	12	7.9
	University	130	86.1
	Graduate (master's/PhD)	8	5.3
Occupation	Midwife	22	14.6
	Housewife	28	18.5
	Nurse	41	27.5
	Teacher	14	9.3
	Other	46	30.5
Income status	Income less than expenses	33	21.9
	Income equal to expenses	103	68.2
	Income greater than expenses	15	9.9
Number of births	None	112	74.2
	One	25	16.6
	Two or more	14	9.3
Mode of birth	None	112	74.2
	Normal	13	8.6
	Cesarean	23	15.2
	Normal and cesarean	3	2.0
Presence of diagnosed illness	Yes (anemia, asthma, thyroid, etc.)	32	21.2
	No	119	78.8
Presence of medication	Yes (euthyrox, thyroid medication, etc.)	23	15.2
	No	128	84.8
Total		151	100

The distribution of correct and incorrect answers given by participants to the questions in the knowledge test is presented in Table 2.

**Table 2:** Distribution of correct and incorrect answers given by participants to the questions in the knowledge test.

Questions	Pre-training incorrect		Pre-training correct		Post-training incorrect		Post-training correct	
	n	%	n	%	n	%	n	%
1*	40	26.7	110	73.3	5	3.3	145	96.7
2*	29	19.3	121	80.7	1	0.7	149	99.3
3*	46	30.7	104	69.3	3	2	147	98
4*	21	14	129	86	19	12.7	131	87.3
5*	10	6.7	140	93.3	3	2	147	98
6*	20	13.3	130	86.7	10	6.7	140	93.3
7*	112	74.7	38	25.3	69	46	81	54
8*	40	26.7	110	73.3	3	2	147	98
9*	19	12.7	131	87.3	15	10	135	90
10*	19	12.7	131	87.3	0	0	150	100
11*	15	10	135	90	7	4.7	143	95.3
12*	62	41.3	88	58.7	13	8.7	137	91.3
13*	59	39.3	91	60.7	31	20.7	119	79.3
14*	58	38.7	92	61,3	14	9.3	136	90.7
15*	44	29.3	106	70.7	4	2.7	146	97.3
16*	65	43.3	85	56.7	23	15.3	127	84.7
17*	33	22	117	78	21	14	129	86
18*	73	48,7	77	51.3	15	10	135	90
19*	64	42,7	86	57.3	8	5.3	142	94.7
20*	36	24	114	76	31	20.7	119	79.3

* 1. Microbiota is the term given to all microorganisms. (T) 2. The formation of microbiota in the human body begins at puberty. (F) 3. The foundation of human microbiota is determined by the mother's gastrointestinal system and the mode of delivery. (T) 4. The concepts of microbiome and microbiota mean the same thing. (F) 5. Probiotics are live microorganisms that regulate gut health. (T) 6. Prebiotics have a supportive role for probiotics. (T) 7. Beneficial microorganisms living in our intestines are called prebiotics. (F) 8. A healthy vaginal flora is dominated by Lactobacilli. (T) 9. A healthy uterine microbiota is dominated by Lactobacilli. (T) 10. Newborns come into contact with beneficial bacteria in the vagina during normal delivery. (T) 11. Babies born via cesarean section have come into contact with beneficial bacteria in the vagina. (F) 12. In children, the microbiota resembles adult microbiota after the age of one. (T) 13. The use of antibiotics in the womb and postpartum increases microbiota diversity in newborns. (F) 14. The mode of delivery does not determine the diversity of infant gut microbiota. (F) 15. The beneficial flora bacterium in breast milk is *Bifidobacterium*. (T) 16. Infants fed with formula have a higher density of *Bifidobacterium* in their intestines compared to those fed with breast milk. (F) 17. In infants born vaginally, the diversity of microbiota from breast milk is less than that of those born via cesarean section. (F) 18. There is no difference in microbiota diversity in the breast milk of mothers who undergo planned cesarean versus emergency cesarean. (T) 19. There is also no different effects on microbiota from hand-expressed versus pump-expressed breast milk. (F) 20. In cases where healthy vaginal microbiota is disrupted, the number of Lactobacilli decreases. (T)

It was determined that there is a statistically significant difference between participants’ knowledge scores before and after the training, with post-training knowledge scores increasing (p<0.05) (Table 3).

**Table 3:** Comparison of participants’ knowledge scores before and after training.

Variables		Pre-training			Post-training			Z (test value)	P
		Median	x	SD	Median	x	SD		
Age	Under 27 years	75.00	72.11	13.14	95.00	90.63	8.82	-7.190	0.000*
	27 years and older	70.00	67.88	13.21	90.00	88.56	9.97	-7.564	0.000*
Education level	Primary school	60.00	60.00	–	80.00	80.00	–	–	–
	High school	65.00	63.33	13.03	85.00	81.67	17.62	-2.856	0.004*
	University	70.00	70.04	13.24	90.00	90.15	8.33	-9.723	0.000*
	Graduate (master's/PhD)	77.50	78.13	11.32	92.50	92.50	4.63	-2.375	0.018*
*Occupation*	Midwife	75.00	71.59	13.75	95.00	92.05	7.97	-4.117	0.000*
	Housewife	60.00	63.04	13.08	90.00	84.82	14.30	-4.502	0.000*
	Nurse	75.00	73.05	11.77	90.00	90.37	7.53	-5.398	0.000*
	Teacher	67.50	65.71	13.13	92.50	88.93	8.81	-3.302	0.001*
	Other	70.00	71.63	13.34	90.00	90.65	7.42	-5.727	0.000*
Mode of birth	None	75.00	70.85	13.03	90.00	90.45	8.37	-9.001	0.000*
	Normal	60.00	60.00	10.80	90.00	86.92	17.14	-3.117	0.002*
	Cesarean	75.00	71.96	14.36	90.00	88.26	7.92	-4.033	0.000*
	Normal and Cesarean	60.00	60.00	0.00	80.00	76.67	5.77	-1.633	0.102
Comparison of total knowledge scores before and after training		70.00	69.87	13.30	90.00	89.54	9.48	-10.412**	0.000*

* p<0.05,

** Wilcoxon test.

## DISCUSSION

Recent studies have largely revealed the significant effects of the microbiota on women's health^2,4,8^. The common feature of studies in the literature that aim to measure individuals’ awareness of microbiota, probiotics, and prebiotics is their goal of recognizing the factors affecting the microbiota in health maintenance^6,7,9,10^. In this context, the scarcity of educational studies in the literature is noteworthy, and increasing research that enables individuals to establish the relationship between microbiota and health could address this existing limitation. This study, conducted with parallel aims, seeks to educate 151 female participants on the importance of microbiota in women's health through various topics (reproductive system infections, fertilization, infertility, pregnancy, childbirth, postpartum period, and newborn health) with diverse variables (age, occupation, etc.). The microbiota knowledge test, consisting of questions related to the topics covered in the training, was administered before and after the education to evaluate the participants’ knowledge levels.

The research design was prepared using examples of planned educational programs related to women's health. In this context, literature on planned reproductive health education given to women that may be relevant to the topic of "microbiota—protection of vaginal health" included in our training program can be provided as case studies ^5,11–13^.

In a study investigating individuals’ microbiota awareness levels, statistical significance was found for educational level and occupational group concerning microbiota awareness scale scores^14^. In parallel with this research, the current study included 151 female participants, and it was determined that the post-education knowledge scores of participants from all occupational groups were higher than their pre-education scores. When examining the pre-education knowledge scores according to participants’ professions, it was found that those in health-related occupations had higher microbiota awareness compared to other groups.

Looking at other studies, there are evaluations of individuals’ knowledge levels regarding microbiota in relation to prebiotics and probiotics. In some studies, variables such as gender, occupation, and education have been used to investigate individuals’ awareness of probiotics, prebiotics, and microbiota concepts, as well as their consumption of probiotic products^2,4,8^. In a study by Khalesi et al, it was determined that among female participants, those with higher education levels used probiotics more^9^. In this research, when examining the education status of the participants, the proportion of women with master's or doctoral degrees was found to be 5.3%. It was determined that women with undergraduate and graduate degrees had higher awareness. The study revealed a proportional relationship between education level and the awareness of the importance of the relationship between microbiota and health. Therefore, it can be recommended to provide necessary education and supportive care regarding microbiota and health for women who are planning pregnancy but do not have a high level of education.

A study assessing the health literacy related to gut microbiota among Italian women included a total of 1,076 women, comprising pregnant women and women with children under the age of two. Researchers found that pregnant women were less likely to recognize the role of breastfeeding in the development of infant gut microbiota compared to non-pregnant women. Additionally, it was reported that participants had a moderate level of knowledge about infant gut microbiota. Among the participants, 97.5% reported an increased interest in gut microbiota^7^. In this study, it was observed that before education, 69.3% of participants correctly answered the question, "the foundation of human microbiota is determined by the mother's gut system and the method of delivery," while after education, this increased to 98%. The findings of this research indicated that the increase in knowledge scores post-education contributed to women's understanding of how the foods consumed during pregnancy influence their infants’ gut microbiota and the preservation of obstetric health.

During the training, the importance of the connection between vaginal health, normal delivery, newborn microbiota health, and maternal milk was explained in detail to the participants. The increase in knowledge levels identified after the training indicates that the participants understood the relationship between microbiota and health. It was determined that the participants learned about the concept of probiotics and grasped their importance after the training.

## CONCLUSION

As a result of the evaluations conducted, it was concluded that this microbiota education program developed and implemented for women addresses all groups. It was determined that the number of correct answers given by participants in the microbiota knowledge test increased after the training. In this context, it is recommended to include microbiota health education in schools, preconception education, childbirth preparation courses, and general health education to raise societal awareness of microbiota.

## Data Availability

The datasets generated and/or analyzed during the current study are available from the corresponding author upon reasonable request.

## References

[1] Aagaard K, Ma J, Antony KM, Ganu R, Petrosino J, Versalovic J (2014). The placenta harbors a unique microbiome. Sci Transl Med.

[2] Nannini G, Amedei A (2024). Women health and microbiota: different aspects of well-being. World J Gastroenterol.

[3] Blaser MJ, Devkota S, McCoy KD, Relman DA, Yassour M, Young VB (2021). Lessons learned from the prenatal microbiome controversy. Microbiome.

[4] Huang JL, Wang X, Yu F, Li MY, Tang YT (2023). Vaginal microbiota abnormalities in women with unexplained infertility and its treatment. Zhonghua Yu Fang Yi Xue Za Zhi.

[5] Dulger D (2021). Raising awareness to add microbiotas to pregnancy nutrition education. J Obstet Gynecol Neonatal.

[6] Atay R, Hacioglu O (2024). Determination of microbiota awareness levels in women planning pregnancy. Rev Assoc Med Bras (1992).

[7] Consales A, Toscano L, Ceriotti C, Tiraferri V, Castaldi S, Giannì ML (2024). From womb to world: mapping gut microbiota-related health literacy among Italian mothers, a cross-sectional study. BMC Public Health.

[8] Silva VF, Refinetti P, Junior JM, Baracat EC, Pacchetti B (2023). Vaginal microbiome and a prospective analysis of vaginal microbiome testing and personalized probiotics supplementation as a beneficial approach to unlock optimal health for women: a narrative review. Eur Gynecol Obstetr.

[9] Khalesi S, Vandelanotte C, Thwaite T, Russell AMT, Dawson D, Williams SL (2021). Awareness and attitudes of gut health, probiotics and prebiotics in Australian adults. J Diet Suppl.

[10] Barqawi HJ, Adra SF, Ramzi HR, Abouaggour MA, Almehairi SK (2021). Evaluating the knowledge, attitudes and practices of the UAE community on microbiota composition and the main factors affecting it: a cross-sectional study. BMJ Open.

[11] Taner A, Demirel Bozkurt O (2021). Impact of the planned reproductive health training provided to visually impaired women on knowledge level and satisfaction. Turkiye Klinikleri J Nurs Sci.

[12] Silva VF, Refinetti P, Vicariotto F, Baracat EC, Soares JM (2024). Oral probiotics and vaginal microbiome in post-menopause women: an opinion for the improvement of natural therapies in gynecology. Rev Assoc Med Bras (1992).

[13] Galhardo CL, Soares JM, Simões RS, Haidar MA, Rodrigues Lima G, Baracat EC (2006). Estrogen effects on the vaginal pH, flora and cytology in late postmenopause after a long period without hormone therapy. Clin Exp Obstet Gynecol.

[14] Kulcu A, Onal O (2022). Microbiota awareness scale validity and reliability study. Med J SDU.

